# Apt19s-functionalized 3D-printed PCL/58S bioactive glass scaffolds *via* thiol–maleimide click chemistry enhance hBMSC adhesion and osteogenic differentiation

**DOI:** 10.1039/d6ra03452g

**Published:** 2026-07-03

**Authors:** Samira Karimian, Ghasem Dini, Negar Nasri, Fereshteh Mahmoodiyan Najafabadi, Shaghayegh Saharkhiz

**Affiliations:** a Department of Nanotechnology, Faculty of Chemistry, University of Isfahan Isfahan 81746-73441 Iran g.dini@sci.ui.ac.ir; b Department of Biotechnology, Faculty of Biological Science and Technology, University of Isfahan Isfahan 81746-73441 Iran

## Abstract

This study presents a novel aptamer-functionalized 3D-printed scaffold designed to promote adhesion and osteogenic differentiation of human bone marrow-derived mesenchymal stem cells (hBMSCs). Composite scaffolds consisting of 55 wt% polycaprolactone (PCL) and 45 wt% 58S bioactive glass (BG) were fabricated using fused deposition modeling (FDM). Surface biofunctionalization was achieved through thiol–maleimide click chemistry to covalently immobilize thiol-terminated Apt19s aptamer. Successful conjugation was confirmed by carbon quantum dot labeling and fluorescence microscopy. *In vitro* evaluations demonstrated that Apt19s-modified scaffolds (scaffold/Mal/Apt) significantly enhanced hBMSC adhesion and spreading, as shown by SEM and cytoskeletal staining. Metabolic activity reached 157.8 ± 3.7% on day 5 (*p* < 0.001), markedly higher than that of the controls. Osteogenic differentiation was substantially accelerated, evidenced by significantly higher mineral deposition (1.92 ± 0.04 AU *vs.* 1.39 ± 0.04 AU, *p* < 0.01) and peak ALP activity of 1.88 ± 0.03 on day 14. Moreover, key osteogenic genes were strongly upregulated, including Runx2 (4.3-fold) and OCN (6.1-fold) by day 28. These findings suggest that the combination of bioactive ion release from 58S BG and aptamer-mediated cell capture creates a favorable microenvironment for osteogenic differentiation *in vitro*.

## Abstract 2

Thiol–maleimide click chemistry immobilized Apt19s aptamer on 3D-printed PCL/58S scaffolds. The Apt-functionalized scaffolds enhanced hBMSC adhesion and osteogenic differentiation, supporting bone tissue engineering.

## Graphical abstract

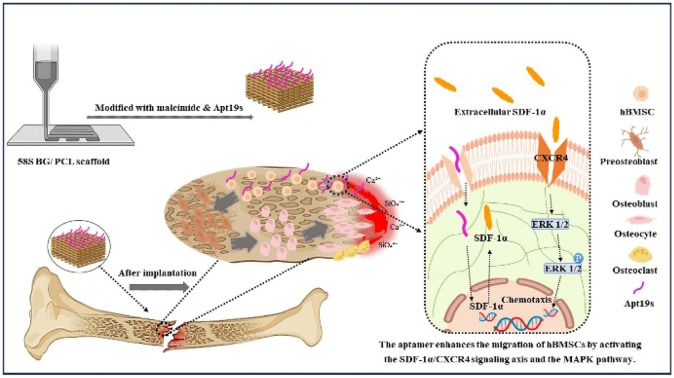

## 1. Introduction

Bone is a highly dynamic tissue that provides structural support and plays critical roles in mineral homeostasis and hematopoiesis.^1^ However, large-scale bone defects often surpass the intrinsic regenerative capacity of native bone, necessitating innovative therapeutic strategies.^2,3^ Bone tissue engineering has emerged as a promising approach, offering three-dimensional scaffolds that guide functional tissue regeneration.^4^ The success of such scaffolds depends on their ability to mimic the native bone extracellular matrix, including both mechanical integrity and biological cues.^5,6^

In this study, a composite scaffold of polycaprolactone (PCL) and 58S bioactive glass (58S BG) nanoparticles was designed. PCL provides tunable biodegradability and mechanical flexibility, while 58S BG enhances bioactivity and osteoinductive capacity through sustained release of calcium and silicate ions.^7,8^ The composite was fabricated *via* fused deposition modeling (FDM) 3D printing, enabling a controlled microstructure with optimized porosity for cell infiltration and nutrient transport.^9,10^

A key limitation of many scaffolds is the lack of specific biological cues on their surfaces.^11^ Growth factors such as bone morphogenetic protein-2 (BMP-2) are potent osteogenic inducers, but their clinical use is limited by short half-life, high cost, instability, and side effects like ectopic ossification.^12,13^ DNA aptamers—short, single-stranded nucleic acid ligands with high target specificity and chemical stability—offer a promising alternative for scaffold biofunctionalization.^14^

**Scheme 1:**
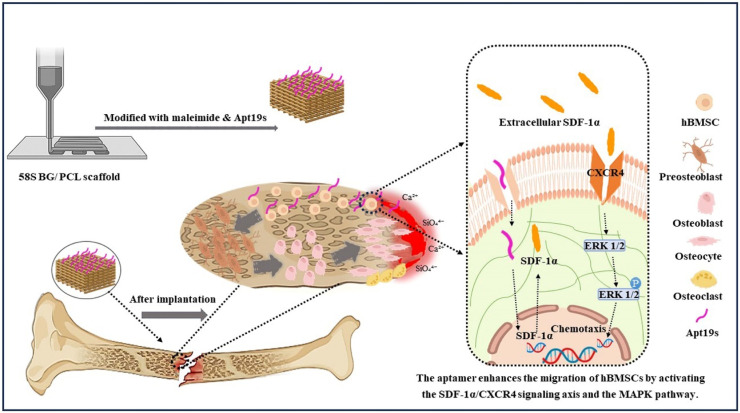
A diagram of a 58S BG/PCL scaffold functionalized with Mal and conjugated with Apt19s for targeted bone regeneration.

While our previous study^15^ demonstrated the potential of Apt19s-functionalized PCL/HA scaffolds for bone tissue engineering, the present work introduces two key innovations beyond that earlier formulation. First, 58S BG replaces hydroxyapatite (HA), providing not only calcium ions but also sustained release of silicate ions (SiO_4_^4−^), which are known to activate osteogenic signaling pathways more effectively than HA alone. Second, the ceramic content is increased from 25 wt% to 45 wt%, creating a more bioactive microenvironment. Here, we hypothesized that replacing HA with 58S BG—which has faster dissolution, higher ionic release, and superior apatite-forming ability—combined with covalent immobilization of Apt19s *via* thiol–maleimide click chemistry, would create a more effective microenvironment for bone regeneration. Therefore, this study aims to develop and evaluate a 3D-printed PCL/58S BG scaffold (45 wt% BG) biofunctionalized with thiol-terminated Apt19s (Scheme 1). We investigate how sustained release of Ca^2+^ and SiO_4_^4−^ ions from the glass phase synergizes with Apt19s-mediated hBMSC adhesion to enhance cell proliferation and osteogenic differentiation. This platform combines FDM 3D printing precision with bio-orthogonal surface functionalization to advance scaffold-based bone repair.

## 2. Materials and methods

### 2.1. Materials

Tetraethyl orthosilicate (TEOS), triethyl phosphate (TEP), and calcium nitrate tetrahydrate were used for the sol–gel synthesis of 58S BG nanoparticles.^16^ Maleimide-terminated polycaprolactone (Mal-PCL, *M*_n_ = 25 000 g mol^−1^) was synthesized *via* ring-opening polymerization (see SI). All other reagents, including potassium hydroxide, EDC, NHS, dithiothreitol (DTT), polycaprolactone (PCL, *M*_w_ = 80 000 g mol^−1^), and staining agents (alizarin red S, DAPI, phalloidin), were purchased from Sigma-Aldrich. Thiol-terminated Apt19s (sequence: 5′-thiol-AAA AAA AAA AGG TCA GAT GAG GAG GGG GAC TTA GGA CTG GGT TTA TGA CCT ATG CGT G-3′) was obtained from Gene Fanavaran Company. hBMSCs were supplied by the Royan Institute (Isfahan, Iran).

### 2.2. Scaffold fabrication *via* 3D printing

3D porous scaffolds (1 × 1 × 0.5 cm^3^, 50 vol% porosity) were fabricated by FDM method using an N1 bioprinter (3DPL®, Iran). A composite containing 45 wt% 58S BG nanoparticles and 55 wt% PCL was prepared and printed following the optimized parameters reported in our previous study,^17^ with adjustment of the ceramic content for the present BG-based formulation.

### 2.3. Surface functionalization of 3D-printed scaffolds

#### 2.3.1. Grafting of maleimide groups

The as-printed scaffolds were subjected to alkaline hydrolysis in 5 M KOH for 2 h to introduce hydroxyl (–OH) and carboxyl (–COOH) functional groups on the surface. After neutralization, the carboxyl groups were activated using EDC/NHS chemistry. Subsequently, Mal-PCL was spin-coated onto the activated surface.^15^ The interaction between Mal-PCL and the activated scaffold surface is primarily physical entrapment and interpenetration, not covalent grafting.

#### 2.3.2. Aptamer immobilization

Thiol-terminated Apt19s was reduced with DTT and purified by dialysis. The activated aptamer was then covalently conjugated to scaffold/Mal *via* thiol–maleimide click chemistry by micro-pipetting, followed by incubation at 4 °C.^15^ The final constructs are designated scaffold/Mal/Apt.

### 2.4. Characterization of aptamer immobilization

Carbon quantum dots (CQDs) were synthesized hydrothermally and conjugated to Apt19s using EDC/NHS chemistry. The Apt/CQD conjugate was immobilized on scaffold/Mal under the same conditions as the unlabeled aptamer. Successful conjugation was verified by fluorescence microscopy (excitation 360 nm, emission 450 nm) as previously reported.^15^

### 2.5. *In vitro* biological evaluation

hBMSCs (passages 3–6) were obtained from the Royan Institute (Isfahan, Iran). The cells were isolated from bone marrow aspirates of healthy donors following written informed consent. All procedures were performed in accordance with the Declaration of Helsinki and approved by the Ethics Committee of the Royan Institute (Isfahan, Iran). The cells were pooled from multiple donors (commercial grade); therefore, detailed donor information is not available. Cells were cultured in high-glucose DMEM supplemented with 10% heat-inactivated FBS and 1% penicillin/streptomycin at 37 °C in a humidified 5% CO_2_ atmosphere. For all assays, cells were seeded onto the scaffolds at a density of 1 × 10^4^ cells per sample.

Cell adhesion was evaluated 24 h after seeding by scanning electron microscopy (SEM). Samples were fixed in 2.5% glutaraldehyde for 2 h, washed five times with sterile water, dehydrated through a graded ethanol series (25%, 50%, 75%, 95%, and 100%), dried in a desiccator for 48 h, and sputter-coated with ∼15 nm gold before imaging.

Metabolic activity was assessed on days 1, 3, and 5 using the MTT assay. Scaffolds were incubated with 1 mL cell suspension (40 000 cells) in 24-well plates. After 24 h, the medium was replaced with 400 µL fresh medium and 100 µL MTT solution (5 mg mL^−1^ in PBS, pH 7.3). Following 3 h incubation, formazan crystals were dissolved in 200 µL DMSO, and absorbance was measured at 570 nm. Three independent biological replicates (*n* = 3) were performed, each with three technical replicates.

Mineralization was quantified on day 14 by alizarin red S staining. Samples were incubated with 40 mM alizarin red S solution (pH 4.2) for 30 min at room temperature, washed with deionized water, and imaged qualitatively. For quantitative analysis, bound dye was extracted using 10% acetic acid, neutralized with ammonium hydroxide, and the absorbance was measured at 405 nm.

Alkaline phosphatase (ALP) activity was measured on days 7, 14, and 21 using *p*-nitrophenyl phosphate as substrate. Samples were washed twice with cold PBS, lysed with 150 µL cold RIPA buffer, shaken in the dark for 60 min, and centrifuged at 14 000 rpm for 15 min. Supernatants were collected, and absorbance was measured at 405 nm.

Gene expression levels of Runx2, ALP, OCN, and OPN were analyzed on days 7, 14, 21, and 28 by quantitative real-time PCR (qRT-PCR) using the ΔΔ*C*_t_ method with GAPDH as the housekeeping gene. Total RNA was extracted using a commercial kit, and cDNA was synthesized with random primers. Each 20 µL qRT-PCR reaction contained 10 ng cDNA, 0.4 µM of each primer, and SYBR Green Master Mix. Primer sequences, amplification efficiencies, and standard curve parameters are provided in SI Table S4.

Cytoskeletal organization was examined on day 3 by fluorescence microscopy after staining with DAPI (nuclei) and Alexa Fluor 488-phalloidin (F-actin). Cells were fixed with 4% paraformaldehyde for 15 min, permeabilized with 0.1% Triton X-100 for 5 min, and stained with phalloidin (1 : 200, 30 min) and DAPI (1 µg mL^−1^, 5 min).

The control group in all experiments consisted of hBMSCs cultured on tissue culture plastic (TCP) without any scaffold. For qRT-PCR analysis, the control group was sampled at day 0 (before osteogenic induction).

### 2.6. Statistical analysis

All quantitative results are expressed as mean ± standard deviation (SD). Three independent biological replicates (*n* = 3) were performed for each experimental condition, where each biological replicate represents a separate scaffold sample seeded with hBMSCs obtained from a different passage. For each biological replicate, three technical replicates were measured, and the values were averaged to minimize measurement error.

Statistical analyses were performed using GraphPad Prism software (version 9.0). Data normality was confirmed using the Shapiro–Wilk test (*p* > 0.05). For multi-time-point assays (MTT, ALP activity, qRT-PCR, and alizarin red quantification), two-way analysis of variance (ANOVA) was performed with scaffold type (three levels: control, scaffold/Mal, and scaffold/Mal/Apt) and time (three or four levels: days 1, 3, 5, or days 7, 14, 21, 28) as fixed factors. Tukey's *post hoc* test was used for multiple comparisons. For single-time-point comparisons, one-way ANOVA with Tukey's *post hoc* test was applied. A *p*-value < 0.05 was considered statistically significant. Significance levels are indicated in the figures as *p < 0.05, **p < 0.01, ***p < 0.001, and ****p < 0.0001. Non-significant comparisons are denoted as “ns”.

For qRT-PCR analysis, gene expression was normalized to the housekeeping gene GAPDH and presented as fold change relative to the control group consisting of hBMSCs cultured on TCP without any scaffold (day 0).

## 3. Results and discussion

### 3.1. Characterization of 58S BG nanoparticles and PCL/BG scaffolds

In this study, 58S BG nanoparticles were synthesized using the sol–gel method, yielding a highly pure amorphous material with chemical and structural characteristics suitable for bone regeneration applications. Characterization analyses confirmed the nanoscale dimensions and spherical morphology of the 58S BG particles, as well as their chemical composition, which closely matched that of the standard 58S powder (Fig. S1a–d; Tables S1 and S2).

A composite scaffold consisting of 45 wt% 58S BG nanoparticles and 55 wt% PCL was fabricated based on a prior optimization study.^17^ The composite demonstrated significantly higher values for compressive strength, yield strength, and compressive modulus than pure PCL, according to mechanical assessments (Fig. S2 and Table S3). Scaffold degradation and bioactivity were assessed over 28 days of immersion in PBS and SBF, respectively. The results demonstrated controlled weight loss, progressive structural breakdown, and the formation of a bone-like apatite layer on the scaffold surface (Fig. S3–S6), confirming its desirable degradability and bioactive potential.

### 3.2. Evaluation of Apt–CQD immobilization on 3D-printed scaffolds

**Fig. 1:**
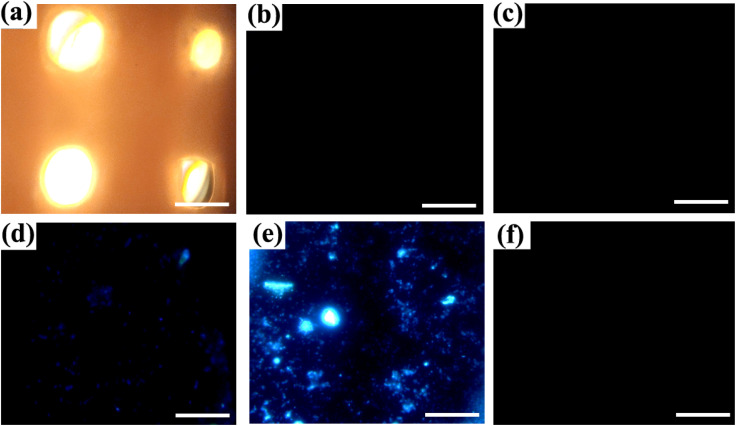
(a) Optical image of the porous 3D-printed scaffold; (b) unmodified scaffold – no fluorescence; (c) scaffold/Mal – no fluorescence; (d) scaffold/Mal + CQDs alone – negligible fluorescence; (e) scaffold/Mal/Apt–CQD – bright homogeneous blue fluorescence; (f) scaffold (no Mal) + Apt–CQD – negligible fluorescence after purification. Scale bar = 200 µm.

A composite scaffold composed of 45 wt% 58S BG nanoparticles and 55 wt% PCL was surface-biofunctionalized with thiol-terminated Apt19s *via* maleimide-mediated thiol–maleimide click chemistry. This bio-orthogonal reaction enables the formation of stable covalent thioether linkages under mild conditions and has been widely used for reliable biomaterial functionalization.^18–23^ Fluorescence microscopy was employed to confirm successful aptamer immobilization using carbon quantum dot-labeled Apt19s (Apt–CQDs). As shown in Fig. 1, the unmodified scaffold (Fig. 1b) and the maleimide-functionalized scaffold without Apt–CQDs (Fig. 1c) exhibited no detectable fluorescence. Incubation of the maleimide-functionalized scaffold with CQDs alone resulted in only negligible fluorescence (Fig. 1d), indicating minimal nonspecific adsorption. In contrast, the scaffold/Mal/Apt–CQD group displayed intense and homogeneous blue fluorescence throughout the porous structure (Fig. 1e), demonstrating successful immobilization of the aptamer. Importantly, the control scaffold lacking maleimide groups but incubated with Apt–CQDs under identical conditions showed no significant fluorescence signal after thorough purification (Fig. 1f). This result confirms that stable attachment of Apt19s occurs predominantly through specific thiol–maleimide click chemistry rather than physical adsorption. All scaffolds were subjected to rigorous purification using a 3 kDa dialysis membrane after each modification step to remove physically adsorbed or unreacted species. Quantitative fluorescence intensity analysis using ImageJ software revealed that the mean fluorescence intensity of scaffold/Mal/Apt was 4.2 ± 0.3-fold higher than that of the CQD-only control and background (*p* < 0.001). The use of CQDs as fluorescent probes provided a highly sensitive and photostable method for visualizing aptamer distribution on the highly porous 3D scaffold surface.^24–26^

### 3.3. Morphological analysis and cell adhesion

**Fig. 2:**
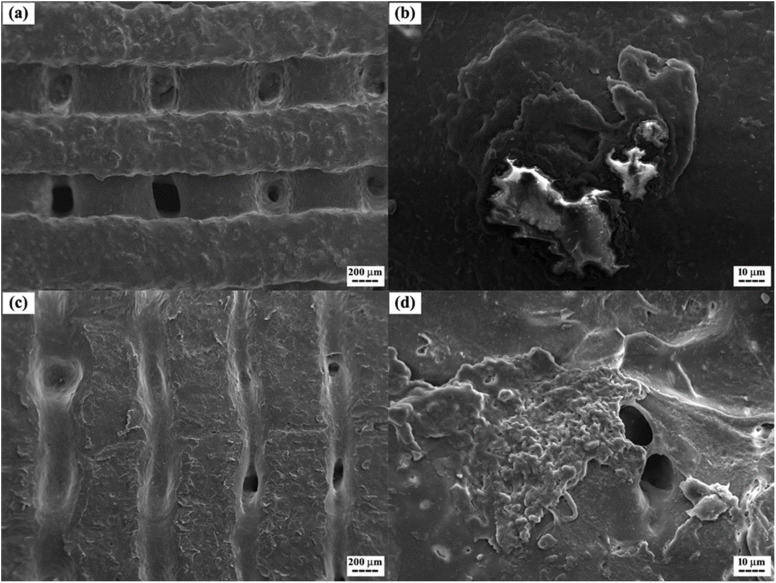
Representative SEM images of hBMSCs seeded on (a and b) scaffold/Mal and (c and d) scaffold/Mal/Apt after one day of *in vitro* culture. Panels (a) and (c) show low magnification, while panels (b) and (d) show high magnification.

SEM analysis (Fig. 2) revealed notable differences in hBMSC adhesion between the two scaffold groups. Cells on scaffold/Mal/Apt (Fig. 2c and d) displayed significantly enhanced spreading, higher cell density, and more pronounced cytoplasmic extensions compared to those on scaffold/Mal (Fig. 2a and b). Quantitative analysis using ImageJ revealed approximately 1.8-fold higher surface coverage on the Apt19s-functionalized scaffolds (48.3 ± 2.1% *vs.* 26.7 ± 1.9%, *p* < 0.01, *n* = 5 independent fields of view per group). These morphological features indicate significantly enhanced cell–scaffold interactions and improved adhesion on the aptamer-functionalized surfaces. Cell adhesion is the critical first step that governs subsequent proliferation, migration, and differentiation. The observed improvement in hBMSC attachment on scaffold/Mal/Apt can be attributed to the synergistic action of maleimide-mediated surface activation and the specific molecular recognition provided by immobilized Apt19s. The aptamers enable targeted binding to cell-surface receptors, thereby activating integrin-mediated signaling pathways that promote cell migration and firm anchorage. These findings are consistent with previous reports demonstrating that functionalization with specific biological ligands substantially strengthens cell–matrix interactions.^27–29^ Collectively, the integration of bioactive aptamers with the structurally optimized 3D-printed scaffold creates a biomimetic microenvironment that effectively supports the initial stages of tissue regeneration.

### 3.4. MTT assay analysis

**Fig. 3:**
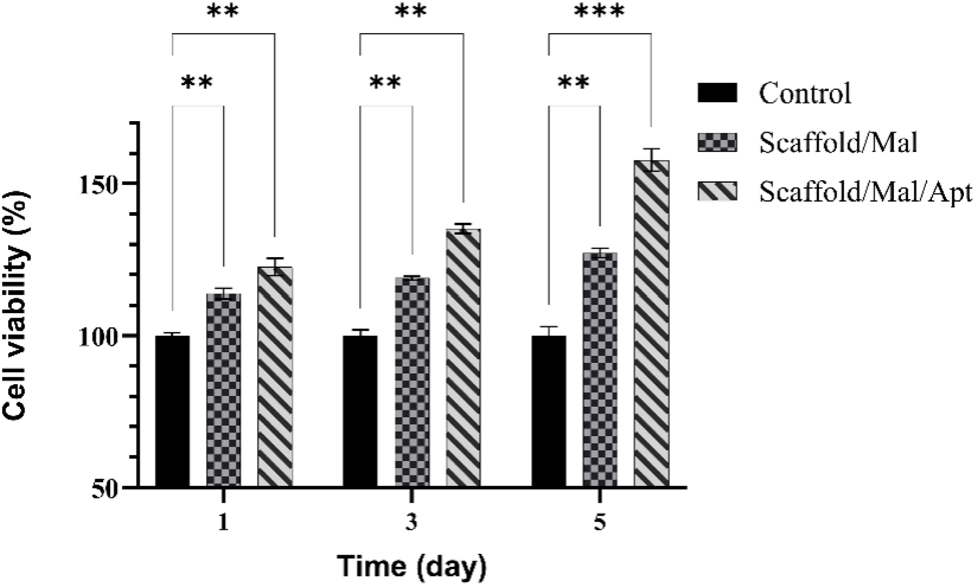
MTT assay results showing cell viability of hBMSCs on days 1, 3, and 5 for the control group (cells on TCP without scaffold), scaffold/Mal, and scaffold/Mal/Apt groups. Data represent mean ± SD (*n* = 3). Significance markers indicate comparison with the control group at the same time point (**p < 0.01, and ***p < 0.001).

Quantitative MTT assay (Fig. 3) demonstrated a clear time-dependent increase in hBMSC metabolic activity on the functionalized scaffolds. On day 1, scaffold/Mal/Apt exhibited higher viability (122.6 ± 2.9%) compared with the control (100.0 ± 1.0%, *p* < 0.01), while scaffold/Mal showed comparable values (113.9 ± 1.7%). By day 3, proliferation was significantly enhanced on scaffold/Mal/Apt (135.2 ± 1.5%, *p* < 0.01 *versus* control), whereas scaffold/Mal displayed only a moderate rise (118.9 ± 0.7%). After 5 days, viability remained elevated in both modified groups, reaching 127.2 ± 1.6% (*p* < 0.01) for scaffold/Mal and a maximum of 157.8 ± 3.7% (*p* < 0.001) for scaffold/Mal/Apt.

These results confirm that aptamer-mediated surface functionalization significantly improves hBMSC viability and proliferation compared with both scaffold/Mal and control groups across all time points. The enhanced metabolic activity arises from the synergistic effect of bioactive ions released from 58S BG (promoting cellular functions) and the specific molecular recognition provided by immobilized Apt19s, which facilitates improved cell signaling and survival pathways.^30–32^ Previous studies have established that the combination of PCL with BGs effectively supports stem cell growth and differentiation.^33–35^ Moreover, Apt19s has been shown to exert no cytotoxic effects on human osteoblasts while enhancing adhesion, proliferation, and maintenance of stemness in rat BMSCs at optimal concentrations.^32,36^ For example, a PCL/small intestine submucosa (SIS) scaffold with dual delivery of rapidly released Apt19s and sustained pBMP2 significantly improved bone regeneration in rat critical-sized calvarial defects without exogenous cell transplantation.^37^

### 3.5. Analysis of hBMSC cytoskeletal arrangement *via* phalloidin–DAPI co-staining

**Fig. 4:**
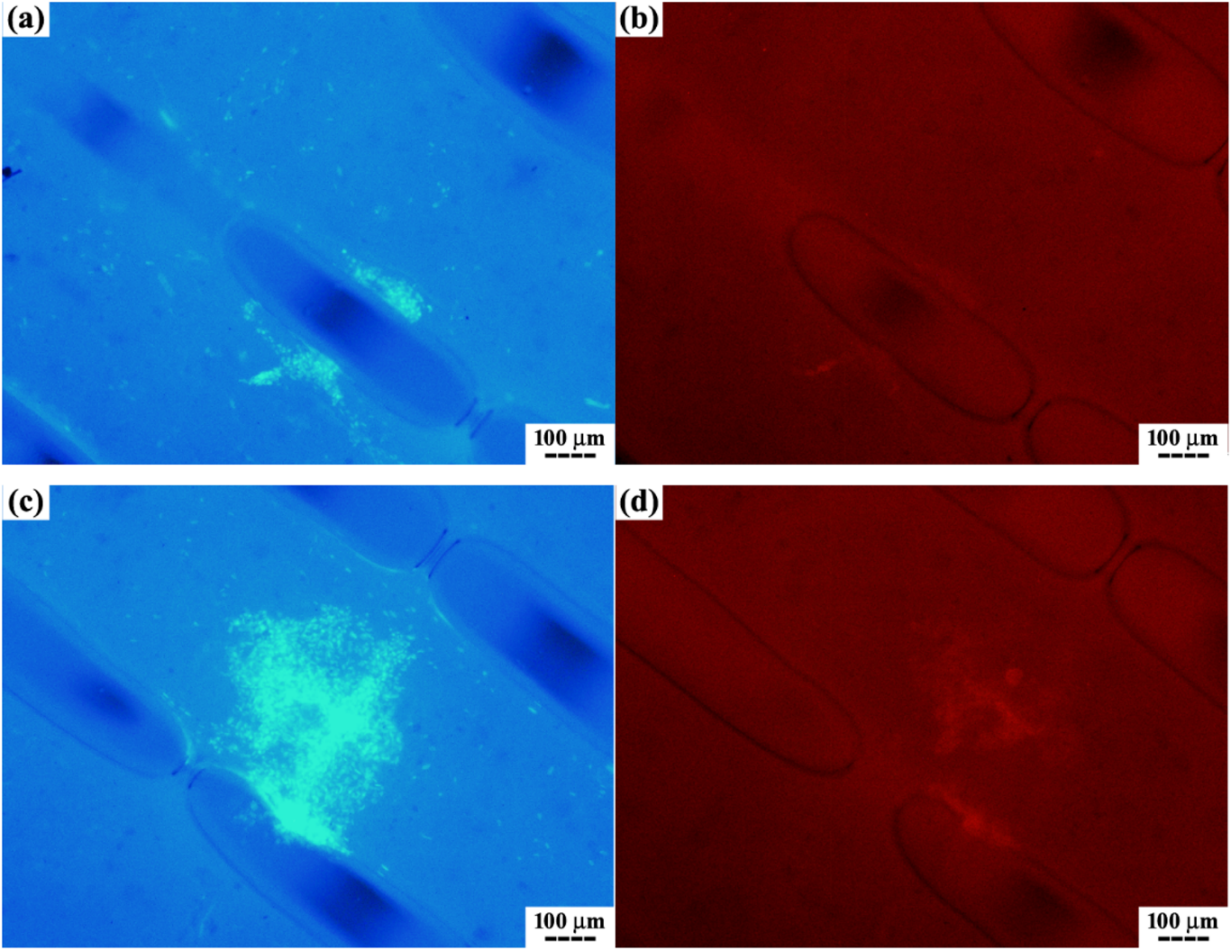
Fluorescence micrographs of hBMSCs cultured for 3 days on (a and b) scaffold/Mal and (c and d) scaffold/Mal/Apt substrates. Nuclear staining with DAPI (a and c) and F-actin visualization with phalloidin (b and d) demonstrate enhanced cytoskeletal organization and increased cell density on the functionalized scaffolds.

Fluorescence microscopy after DAPI/phalloidin co-staining revealed clear differences in the cytoskeletal organization of hBMSCs after 3 days of culture (Fig. 4). Cells cultured on scaffold/Mal/Apt (Fig. 4c and d) exhibited well-developed F-actin stress fibers, more elongated morphology, and higher cell density compared with those on scaffold/Mal (Fig. 4a and b). Quantitative image analysis of DAPI-stained nuclei using ImageJ software demonstrated significantly higher cell density on scaffold/Mal/Apt (176 ± 12 cells per mm^2^) compared to scaffold/Mal (94 ± 9 cells per mm^2^) (*p* < 0.01, *n* = 5 independent fields per group), corresponding to an approximately 1.9-fold increase.

These morphological improvements are consistent with the higher metabolic activity observed in the MTT assay and further confirm the good cytocompatibility of the aptamer-functionalized scaffolds. The enhanced cell–scaffold interaction is mediated by optimized integrin binding and activation of adhesion-related signaling cascades triggered by immobilized Apt19s. Such improvements align with previous studies on aptamer-mediated biointerfaces, where nucleic acid ligands have been shown to strengthen cell–matrix interactions through specific molecular recognition.^27,29,38^ For instance, Apt19s-modified 3D PCL/BG scaffolds promoted rBMSC attachment and maintained structured cytoskeletal architecture,^39^ while Apt19s-loaded dynamic hydrogels enhanced MSC adhesion, synergistically supporting osteogenic differentiation.^40^

### 3.6. Detection of calcium-rich matrix *via* alizarin red assay

**Fig. 5:**
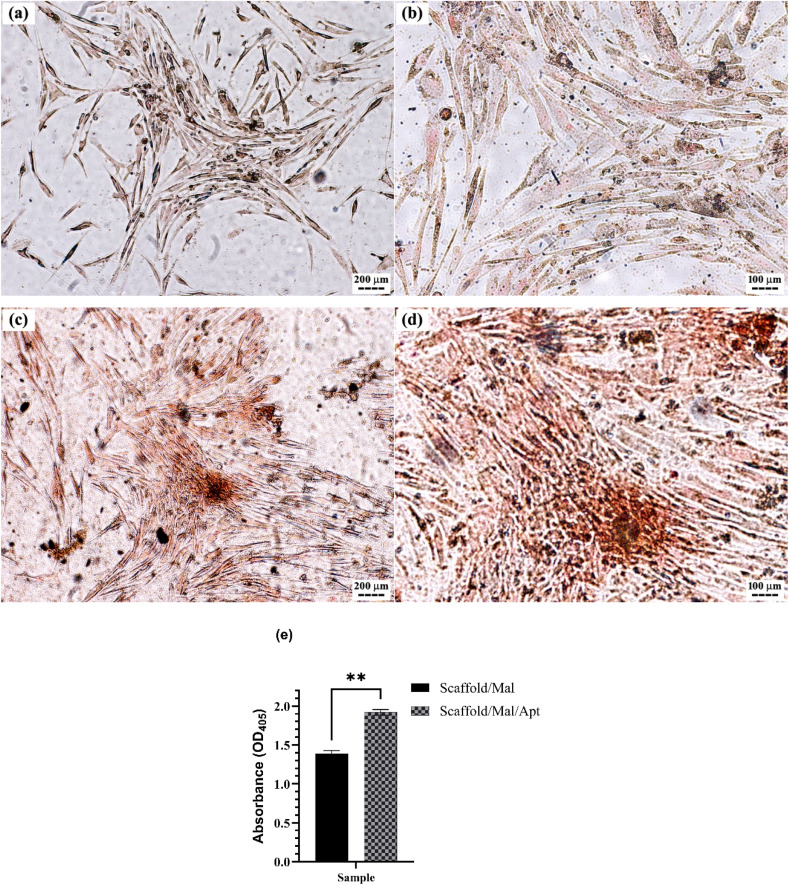
Evaluation of calcium deposition *via* alizarin red staining in hBMSCs cultured on (a and b) scaffold/Mal and (c and d) scaffold/Mal/Apt substrates at different magnifications. (e) Quantitative spectrophotometric analysis of extracted alizarin red dye after 14 days. Data represent mean ± SD (*n* = 3, **p < 0.01 *vs.* scaffold/Mal).

Alizarin red S staining after 14 days of culture (Fig. 5) demonstrated significantly enhanced matrix mineralization on the aptamer-functionalized scaffolds. Scaffold/Mal/Apt constructs exhibited extensive crimson staining with densely distributed mineralized nodules (Fig. 5c and d), whereas scaffold/Mal samples showed only limited pink staining with sparse and isolated calcium deposits (Fig. 5a and b). Quantitative analysis confirmed significantly higher calcium deposition on scaffold/Mal/Apt (1.92 ± 0.04 AU) compared with scaffold/Mal (1.39 ± 0.04 AU), representing an approximately 1.4-fold increase (*p* < 0.01). This substantial improvement in mineralization is attributed to the osteoinductive properties of the covalently tethered Apt19s, which promote osteogenic commitment of hBMSCs through precise molecular recognition. These observations are consistent with previous findings showing that optimal concentrations of Apt19s enhance cellular adhesion and mineral matrix production in rBMSCs.^41^ Similarly, multifunctional Apt19s-based scaffolds have been shown to effectively attach MSCs and accelerate their osteogenic differentiation and biomineralization.^42^

### 3.7. Analysis of alkaline phosphatase activity

**Fig. 6:**
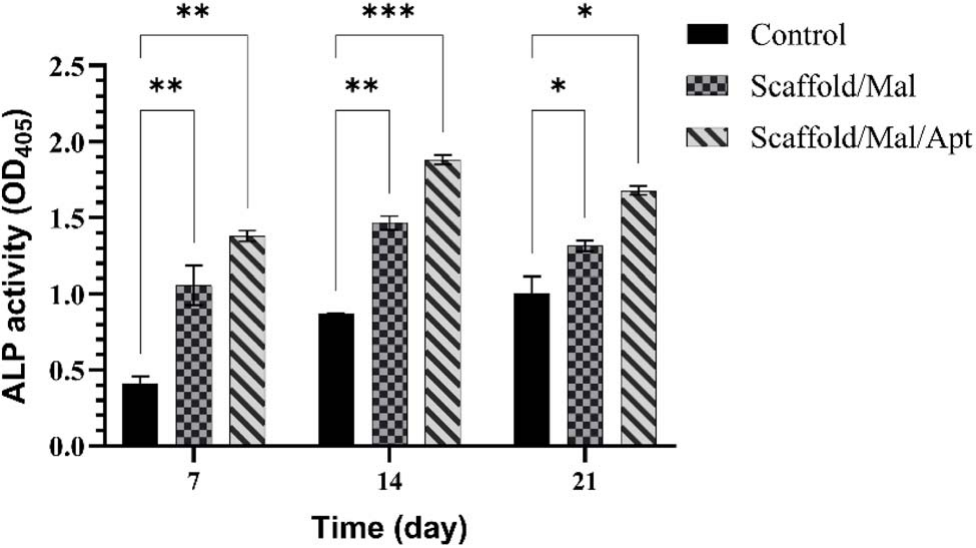
Alkaline phosphatase activity of hBMSCs cultured on scaffold/Mal and scaffold/Mal/Apt substrates on days 7, 14, and 21 (control: hBMSCs on TCP without scaffold). Data represent mean ± SD (*n* = 3). *p < 0.05, **p < 0.01, ***p < 0.001 *vs.* control group at the same time point.

ALP activity showed clear time-dependent changes across the experimental groups (Fig. 6). At day 7, scaffold/Mal/Apt exhibited substantially higher ALP levels (1.38 ± 0.04) compared with scaffold/Mal (1.06 ± 0.13) and the control (0.41 ± 0.05), indicating early osteogenic commitment. By day 14, ALP activity continued to rise, with scaffold/Mal/Apt reaching a peak of 1.88 ± 0.03, significantly higher than scaffold/Mal (1.47 ± 0.05) and control (0.87 ± 0.02). On day 21, values converged, with scaffold/Mal/Apt (1.68 ± 0.03) approaching the control level (1.00 ± 0.11). Overall, surface functionalization preferentially accelerated early-stage osteogenic maturation, with the most pronounced effects observed within the first two weeks of culture.

This enhanced and accelerated ALP activity is attributed to the synergistic combination of bioactive ions (Ca^2+^ and SiO_4_^4−^) released from the 58S BG component, which provide essential osteogenic cues, and the stable covalent immobilization of Apt19s, which ensures sustained bioactivity and targeted stem cell interactions.^30,33^ The resulting microenvironment significantly amplifies early osteogenic differentiation. While several aptamer-functionalized scaffolds have previously shown improved mineralization and cell growth, one study using BMSC-aptamer hydrogels reported no significant increase in ALP activity compared with controls.^43^ The superior performance observed here highlights the critical role of both scaffold composition (58S BG/PCL) and the thiol–maleimide conjugation strategy in achieving substantial early osteogenic induction.

### 3.8. Quantitative analysis of osteogenic gene expression

**Fig. 7:**
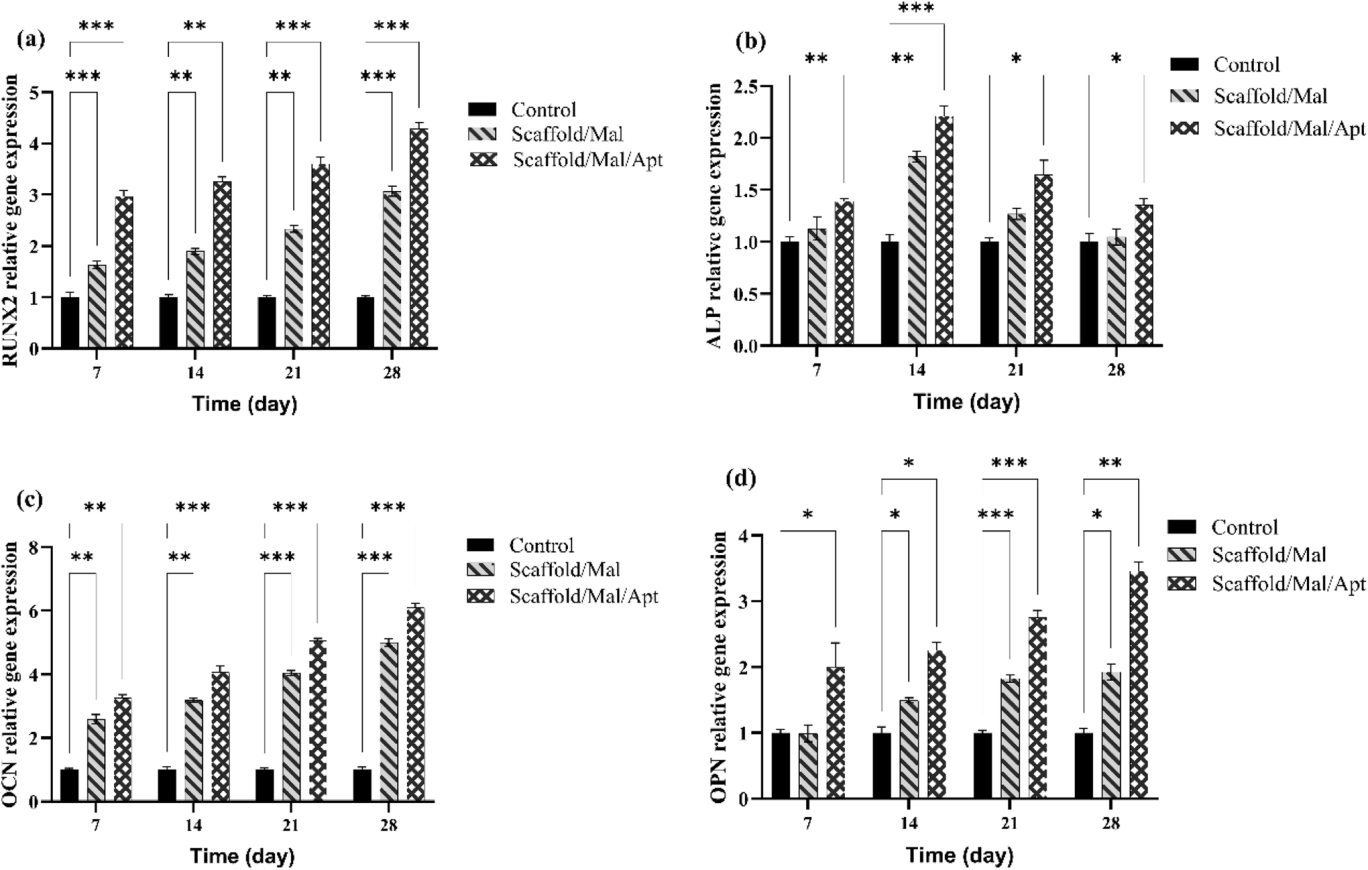
Relative mRNA expression of osteogenic markers in hBMSCs cultured on control (TCP without scaffold, day 0), scaffold/Mal, and scaffold/Mal/Apt groups. Expression levels of (a) OPN, (b) RUNX2, (c) ALP, and (d) OCN were assessed on days 7, 14, 21, and 28. Data represent mean ± SD (*n* = 3). *p < 0.05, **p < 0.01, ***p < 0.001 *vs.* control group.

qRT-PCR analysis was performed over 28 days to evaluate the expression of key osteogenic markers (Runx2, ALP, OCN, and OPN) in hBMSCs cultured on the scaffolds (Fig. 7). In scaffold/Mal/Apt, Runx2 (Fig. 7a), the master regulator of osteogenesis, showed rapid and sustained upregulation, increasing 3.0-fold by day 7 (*p* < 0.001) and reaching 4.3-fold by day 28 (*p* < 0.001). ALP expression (Fig. 7b) displayed its characteristic early-peak pattern, attaining a maximum of 2.2-fold at day 14 (*p* < 0.001), consistent with accelerated transition from proliferation to differentiation. Late-stage markers also exhibited strong enhancement: OCN (Fig. 7c) increased up to 6.1-fold by day 28 (*p* < 0.001), and OPN (Fig. 7d) rose to 3.5-fold (*p* < 0.01), indicating substantial matrix maturation and mineralization.

This sequential activation—from early transcriptional regulators (Runx2) through intermediate (ALP) to late-stage matrix proteins (OCN and OPN)—demonstrates that Apt19s functionalization drives a complete and accelerated osteogenic cascade. The superior gene expression profile in scaffold/Mal/Apt stems from three synergistic design elements: the selective molecular recognition of Apt19s for targeted hBMSC adhesion, stable thiol–maleimide covalent immobilization ensuring prolonged aptamer presentation, and the biomimetic properties of the PCL/BG composite that closely mimic native bone extracellular matrix.

These results align with previous studies showing that Apt19s-functionalized 3D-printed MBG scaffolds enhance MSC migration and osteogenic differentiation *via* upregulation of SDF-1α and activation of SDF-1α/CXCR4 and MAPK signaling pathways, leading to increased expression of ALP, OCN, BMP-2, and OPN.^36,44^ The consistent upregulation pattern observed here across different scaffold compositions (including 58S BG) further confirms the substantial osteoinductive capacity of Apt19s. The pronounced effect is attributed to covalent stabilization of the aptamer, optimal surface conjugation density provided by maleimide chemistry, and the sustained release of bioactive ions (calcium and silicate) from 58S BG nanoparticles, which collectively activate osteogenic signaling pathways and create a favorable microenvironment for bone matrix deposition.

The superior osteogenic gene expression profile observed in the scaffold/Mal/Apt group is driven by the specific molecular recognition of Apt19s. This 49-nucleotide DNA aptamer, selected *via* SELEX, binds with high affinity to membrane proteins on mesenchymal stem cells, promoting their specific adhesion and activating endogenous regenerative pathways.^45,46^ Specifically, Apt19s upregulates stromal cell-derived factor-1α (SDF-1α) expression and secretion, which in turn activates the SDF-1α/CXCR4 axis and downstream MAPK signaling (including p38 and ERK pathways). These cascades enhance stem cell migration and osteogenic differentiation.^47^ Although SDF-1α is known to potentiate BMP-2-induced osteogenesis through Smad1/5 and Erk1/2 phosphorylation, the present study did not directly measure BMP-2 expression or pathway phosphorylation; therefore, potential crosstalk remains to be confirmed in future work.^48,49^

Collectively, these findings demonstrate that the covalent immobilization of Apt19s on 58S BG/PCL scaffolds creates an efficient osteoinductive platform. The integration of aptamer-mediated molecular recognition with the ionic stimulation provided by BG offers a dual mechanism that markedly accelerates osteogenic differentiation and surpasses conventional single-factor approaches. This combination of advanced surface engineering and biomimetic scaffold design represents a potential strategy for scaffold-based bone regeneration.

### 3.9. Comparison with the previously developed PCL/HA scaffold

When compared with our previously reported Apt19s-functionalized PCL/HA scaffold fabricated under similar conditions,^15^ the present PCL/58S BG formulation showed enhanced osteogenic performance under the tested conditions. This improvement may be partially attributed to the different dissolution kinetics and bioactivity profile of 58S BG compared to HA. The faster release of bioactive ions (SiO_4_^4−^ and Ca^2+^) from 58S BG, combined with the higher ceramic content (45 wt% *versus* 25 wt% HA), appears to contribute to a more favorable osteoinductive microenvironment in this study.

Specifically, hBMSC metabolic activity on day 5 increased from 145.3 ± 2.5% to 157.8 ± 3.7%, mineral deposition rose from 1.47 ± 0.03 AU to 1.92 ± 0.04 AU, and peak ALP activity improved from 1.51 ± 0.16 to 1.88 ± 0.03. At the gene expression level, Runx2 and OCN reached 4.3-fold and 6.1-fold upregulation by day 28, respectively, compared to 3.9-fold and 5.86-fold in the HA-based scaffold. These results suggest that the combination of rapid ionic signaling from 58S BG and targeted Apt19s-mediated stem cell adhesion may represent a promising alternative platform for bone regeneration. However, direct comparative studies under identical conditions are needed to confirm these observations.

## 4. Conclusion

This study developed an aptamer-functionalized 3D-printed scaffold composed of 55 wt% PCL and 45 wt% 58S BG, surface-modified with thiol-terminated Apt19s through thiol–maleimide click chemistry. The resulting bioactive platform enhanced hBMSC adhesion, spreading, and cytoskeletal organization compared to non-functionalized controls. Furthermore, it promoted cell metabolic activity (157.8 ± 3.7% on day 5), supported osteogenic differentiation, and increased mineral deposition (1.92 ± 0.04 AU *vs.* 1.39 ± 0.04 AU in scaffold/Mal). These improvements were accompanied by elevated expression of key osteogenic markers, with Runx2 and OCN reaching 4.3-fold and 6.1-fold upregulation by day 28, respectively.

The enhanced *in vitro* performance of the PCL/58S BG scaffold compared to our previous PCL/HA formulation may be attributed to the dual ionic signaling (Ca^2+^ and SiO_4_^4−^) from 58S BG. This dual-action strategy creates a favorable microenvironment for hBMSC osteogenic differentiation *in vitro*.

While the *in vitro* results are promising, this study has certain limitations. The selectivity of Apt19s-mediated effects was not fully validated using scrambled aptamer or physical adsorption controls, although the specific binding of Apt19s to mesenchymal stem cells has been previously established, and the absence of bioactivity on control scaffolds (without aptamer) supports the observed specificity. Additionally, aptamer immobilization was primarily confirmed by fluorescence imaging, and direct surface chemical characterization after each functionalization step was not performed. FTIR analysis was attempted, but the low surface density of the conjugated aptamer resulted in peaks below the detection limit.

It should be noted that these findings are based on *in vitro* experiments, and further *in vivo* studies are required to evaluate the actual bone regeneration efficacy of this scaffold system. Future research will focus on long-term scaffold degradation and biocompatibility, optimization of aptamer surface density, elucidation of the detailed signaling pathways involved, inclusion of appropriate specificity controls, and evaluation of bone regeneration efficacy in critical-sized defect animal models.

## Conflicts of interest

The authors declare that the research was conducted in the absence of any commercial or financial relationships that could be construed as a potential conflict of interest.

## Supplementary Material

RA-OLF-D6RA03452G-s001

## Data Availability

The data presented in this study are available on request from the corresponding author. The data supporting this article have been included as part of the supplementary information (SI). Supplementary information is available. See DOI: https://doi.org/10.1039/d6ra03452g.

## References

[cit1] Manzini B. M., Ribeiro M. L. M., Yoshito N. P. (2021). J. Biosci..

[cit2] Santoro A., Voto A., Fortino L., Guida R., Laudisio C., Cillo M., D'Ursi A. M. (2025). Int. J. Mol. Sci..

[cit3] De Pace R., Molinari S., Mazzoni E., Perale G. (2025). J. Clin. Med..

[cit4] Stahl A., Yang Y. P. (2021). Tissue Eng., Part B.

[cit5] Greening T. (1998). Med. Educ. Online.

[cit6] Xia B., Deng Y., Lv Y., Chen G. (2021). Biomater. Sci..

[cit7] Goldberg D. (1995). J. Environ. Polym. Degrad..

[cit8] Meng L., Zhao P., Jiang Y., You J., Xu Z., Yu K., Boccaccini A. R., Ma J., Zheng K. (2024). Acta Biomater..

[cit9] Naveed N. (2021). Mater. Technol..

[cit10] Winarso R., Anggoro P. W., Ismail R., Jamari J., Bayuseno A. P. (2022). Heliyon.

[cit11] Causa F., Netti P. A., Ambrosio L. (2007). Biomaterials.

[cit12] Oryan A., Alidadi S., Moshiri A., Bigham-Sadegh A. (2014). BioFactors.

[cit13] Qi J., Wu H., Liu G. (2024). Cell Transplant..

[cit14] Abpeikar Z., Alizadeh A. A., Rezakhani L., Ramezani V., Goodarzi A., Safaei M. (2023). Mol. Biotechnol..

[cit15] Doustmohammadi M., Nasri N., Saharkhiz S., Dini G., Najafabadi F. M., Sanyal A. (2025). Results Eng..

[cit16] Bui X. V., Dang T. H. (2019). Process. Appl. Ceram..

[cit17] Soleymani M., Moslemi S., Dini G., Ejeian F., Najafinezhad A. (2026). Sci. Rep..

[cit18] Martínez-Jothar L., Doulkeridou S., Schiffelers R. M., Torano J. S., Oliveira S., van Nostrum C. F., Hennink W. E. (2018). J. Controlled Release.

[cit19] Wang X., Huang Y., Song X., Guo L., Chen G., Yang L., Chen C., Gong X. (2023). J. Nat. Fibers.

[cit20] Kim J., Choi Y., Park J., Lee H. Y., Choi J. (2024). Biotechnol. Bioprocess Eng..

[cit21] Magalhães M. V., Débera N., Pereira R. F., Neves M. I., Barrias C. C., Bidarra S. J. (2024). Carbohydr. Polym..

[cit22] Cengiz N., Gevrek T. N., Sanyal R., Sanyal A. (2020). Bioconjugate Chem..

[cit23] Gober I. N., Sharan R., Villain M. (2023). J. Pept. Sci..

[cit24] Ding Y., Ling J., Wang H., Zou J., Wang K., Xiao X., Yang M. (2015). Anal. Methods.

[cit25] Yazdian F. , in Aptamers Engineered Nanocarriers for Cancer Therapy, Woodhead Publishing, 2023, pp. 295–315

[cit26] Kim D., Yoo S. (2021). Chemosensors.

[cit27] Yang Z., Zhao T., Gao C., Cao F., Li H., Liao Z., Fu L., Li P., Chen W., Sun Z., Jiang S. (2021). ACS Appl. Mater. Interfaces.

[cit28] Li H., Zhao T., Cao F., Deng H., He S., Li J., Liu S., Yang Z., Yuan Z., Guo Q. (2022). Bioeng. Transl. Med..

[cit29] Hsu Y. W., Ma L., Tang Y., Li M., Zhou C., Geng Y., Zhang C., Wang T., Guo W., Li M., Wang Y. (2025). J. Mater. Chem. B.

[cit30] Ducheyne P. (1999). MRS Online Proc. Libr..

[cit31] Najafabadi F. M., Karbasi S., Benisi S. Z., Shojaei S., Poursamar S. A., Azadani R. N. (2023). Mater. Chem. Phys..

[cit32] Gómez-Cerezo N., Lozano D., Salinas A. J., Vallet-Regí M. (2026). Adv. Healthcare Mater..

[cit33] Larranaga A., Alonso-Varona A., Palomares T., Rubio-Azpeitia E., Aldazabal P., Martin F. J., Sarasua J.-R. (2015). J. Biomed. Mater. Res., Part A.

[cit34] Ding Y., Li W., Müller T., Schubert D. W., Boccaccini A. R., Yao Q., Roether J. A. (2016). ACS Appl. Mater. Interfaces.

[cit35] Keothongkham K., Charoenphandhu N., Thongbunchoo J., Suntornsaratoon P., Krishnamra N., Tang I. M., Pon-On W. (2017). Mater. Sci. Eng. C.

[cit36] Luo Z. W., Liu Y. W., Rao S. S., Yin H., Huang J., Chen C. Y., Hu Y., Zhang Y., Tan Y. J., Yuan L. Q., Chen T. H. (2019). Nanoscale.

[cit37] Sun T., Meng C., Ding Q., Yu K., Zhang X., Zhang W., Tian W., Zhang Q., Guo X., Wu B., Xiong Z. (2021). Bioact. Mater..

[cit38] Ren Y., Fan L., Alkildani S., Liu L., Emmert S., Najman S., Rimashevskiy D., Schnettler R., Jung O., Xiong X., Barbeck M. (2022). Int. J. Mol. Sci..

[cit39] Wang Z., Lao A., Huang X., Zhou Y., Shen S. G., Lin D. (2024). Adv. Funct. Mater..

[cit40] He S., Zhao H., Chen M., Li M., Wu Y., Chen H., Zheng Y., Luo Z., Cai K., Hu Y. (2025). Adv. Healthcare Mater..

[cit41] Wang X., Zheng X., Duan Y., Ma L., Gao C. (2019). ACS Appl. Mater. Interfaces.

[cit42] Jin Y., Huang Y., Wang J., Zhou X., Chen J., Ding W., Jia Z., Xu Y. (2025). Burns Trauma.

[cit43] Gong J. S., Zhu G. Q., Zhang Y., Chen B., Liu Y. W., Li H. M., He Z. H., Zou J. T., Qian Y. X., Zhu S., Hu X. Y. (2023). Mater. Today Bio.

[cit44] Zhang R., Pan X., Huang Z., Weber G. F., Zhang G. (2011). PLoS One.

[cit45] Shi S., Liao H., Lu W., Chen T., Sun Y., Lin Y. (2025). Adv. Funct. Mater..

[cit46] Lai B. Q., Wu R. J., Wu C. R., Yu H. Y., Xu J., Yang S. B., Chen Z. H., Li X., Guo Y. N., Yang Y., Che M. T. (2025). Mater. Today Bio.

[cit47] Rodríguez-Carballo E., Gámez B., Ventura F. (2016). Front. Cell Dev. Biol..

[cit48] Qin H., Zhao X., Hu Y. J., Wang S., Ma Y., He S., Shen K., Wan H., Cui Z., Yu B. (2021). BioMed Res. Int..

[cit49] Xiao M., Yao B., Zhang B. D., Bai Y., Sui W., Wang W., Yu Q. (2019). Exp. Cell Res..

