# Supplementary material for: Apt19s-functionalized 3D-printed PCL/58S bioactive glass scaffolds via thiol–maleimide click chemistry enhance hBMSC adhesion and osteogenic differentiation
Source: RSC Adv. 2026 Jul 3;16(35):36387–96. doi: 10.1039/d6ra03452g (PMC13330724; doi:10.1039/d6ra03452g)
Supplement: RA-016-D6RA03452G-s001 [file RA-016-D6RA03452G-s001.pdf]

Supplementary Information (SI) for RSC Advances.
This journal is © The Royal Society of Chemistry 2026

# Apt19s-Functionalized 3D-Printed PCL/58S Bioactive Glass Scaffolds via Thiol-Maleimide Click Chemistry Enhance hBMSC Adhesion and Osteogenic Differentiation

Samira Karimiyan[1], Ghasem Dini[1]*, Negar Nasri[2], Fereshteh Mahmoodiyan Najafabadi[1], Shaghayegh Saharkhiz[2]

1) Department of Nanotechnology, Faculty of Chemistry, University of Isfahan, Isfahan 81746-73441, Iran.
2) Department of Biotechnology, Faculty of Biological Science and Technology, University of Isfahan, Isfahan, 81746-73441, Iran.

*** Corresponding author:**

Ghasem Dini (Ph.D., Associate Professor), Email: g.dini@sci.ui.ac.ir

## 1. Synthesis and Characterization of 58S BG Powder

The 58S BG powder (nominal composition: 58 wt.% $SiO_2$, 33 wt.% CaO, 9 wt.% $P_2O_5$; corresponding molar composition: 60 mol% $SiO_2$, 36 mol% CaO, 4 mol% $P_2O_5$) was synthesized using a sol-gel processing route. For the preparation of 5 g of final powder, 10.07 g of TEOS was first mixed with 10 mL of nitric acid under continuous magnetic stirring. After 20 minutes, 1.15 g of TEP was added to the solution. In a separate container, 6.95 g of calcium nitrate tetrahydrate was fully dissolved in 12 mL of deionized water under agitation. Both precursor solutions were stirred for 24 hours to ensure complete hydrolysis of the silicon- and phosphorus-containing components. The hydrolyzed TEOS/TEP solution was then gradually added to the calcium nitrate solution and continuously stirred for an additional 4 hours, resulting in a transparent sol. The sol was allowed to age at room temperature until gelation occurred. The formed gel was subsequently dried, further aged, and calcined at 600 °C for 3 hours. The calcined product was manually ground using an agate mortar and pestle and then sieved through a 200-mesh screen to obtain fine, uniformly sized particles [1, 2].

XRF analysis (Table S1) confirmed that the chemical composition of the synthesized powder closely matched the theoretical 58S BG formula. The XRD pattern (Fig. S1a) verified the amorphous structure of the material, displaying a characteristic broad halo at approximately $2\theta = 15°$ with no detectable crystalline peaks. SEM micrograph (Fig. S1b) revealed semi-spherical nanoparticles with rough surface morphology and an average size of approximately 20 nm, although partial agglomeration was evident. DLS size analysis (Fig. S1c) indicated an average particle diameter of approximately 50 nm, which shows reasonable agreement with the SEM observations, considering the hydrodynamic nature of DLS measurements. BET analysis (Fig. S1d and Table S2) further demonstrated favorable textural properties, including a specific surface area of 97 $m^2/g$, an average pore diameter of 8 nm, and a total pore volume of 0.3 $cm^3/g$.

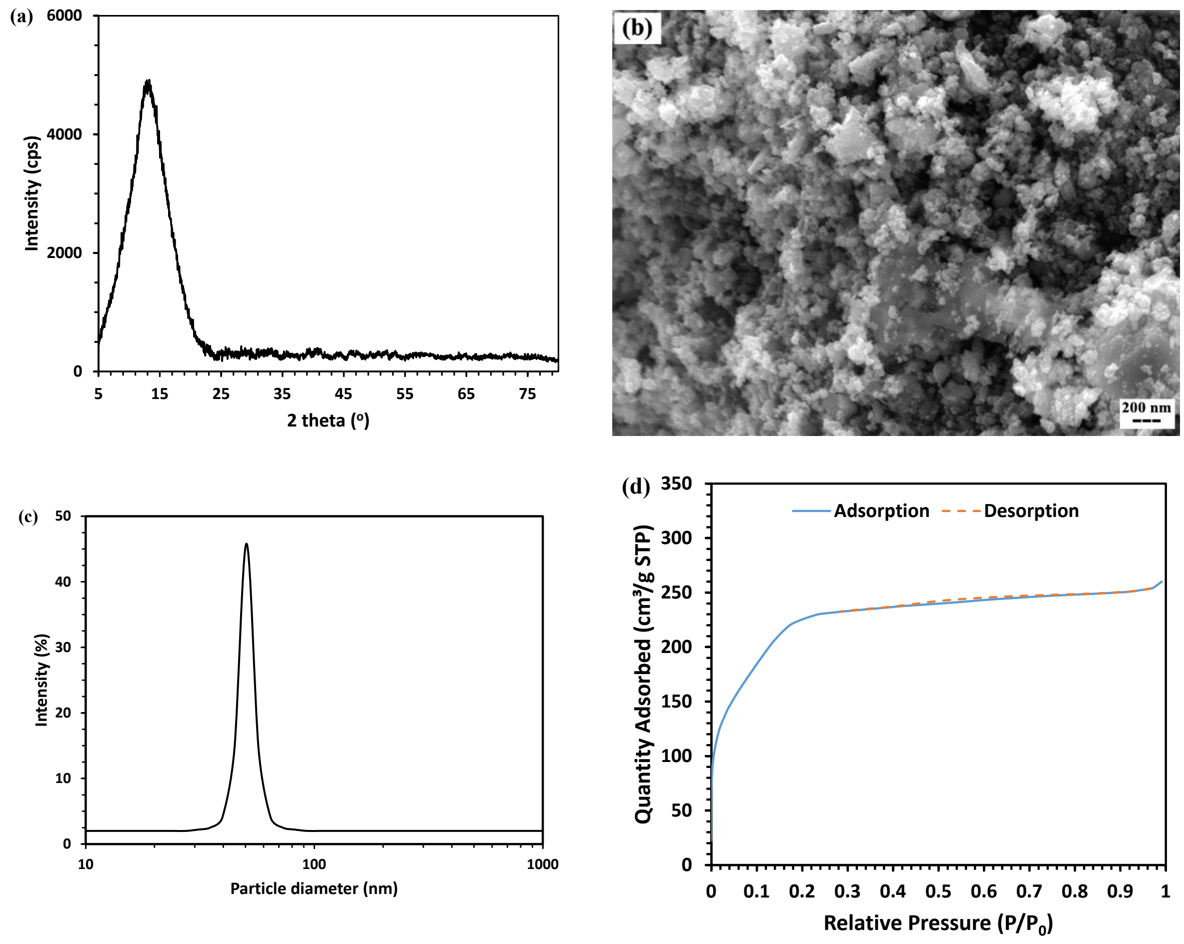


**Figure S1.** (a-d) XRD pattern, SEM micrograph, DLS result, and nitrogen adsorption/desorption isotherm of 58S BG nanoparticles synthesized in this study, respectively.

**Table S1.** Chemical composition of the synthesized 58S BG powder.

| Compound | $SiO_2$ | CaO | $P_2O_5$ | Traces |
|---|---|---|---|---|
| Content (wt. %) | 54.5 | 32.3 | 12.9 | <0.3 |

**Table S2.** Textural properties of 58S BG powder analyzed by BET.

| Powder | Specific surface area ($m^2/g$) | Average pore diameter (nm) | Total pore volume ($cm^3/g$) |
|---|---|---|---|
| BG | 97 | 8 | 0.31 |

## 2. Fabrication and Characterization of 3D-Printed 58S BG/PCL Scaffolds

Composite materials containing 45 wt.% 58S BG nanoparticles within a PCL matrix were prepared. The 58S BG powder was first sieved through a 200-mesh screen to ensure uniform particle size. PCL was dissolved in chloroform under continuous magnetic stirring until complete dissolution. The

predetermined amount of bioactive glass nanoparticles was gradually added to the polymer solution with constant agitation, followed by ultrasonic homogenization to ensure uniform particle dispersion. The mixture was then subjected to solvent evaporation under magnetic stirring for 24 hours. After complete solvent removal, the solidified composite was collected and sectioned for further processing. Three-dimensional scaffolds were fabricated using an N1 bioprinter (3DPL, Iran) based on the fused deposition modeling technique. Scaffold structures were designed as cubic architectures with approximately 50% porosity using G-code programming. Scaffolds composed of 45 wt.% 58S BG and 55 wt.% PCL, with final dimensions of approximately 1 × 1 × 0.5 $cm^3$, were evaluated using compressive testing on a 2T SANTAM mechanical testing system. The incorporation of 45 wt.% 58S BG significantly enhanced the mechanical performance of the composite, increasing the compressive modulus to 0.84 ± 0.04 GPa and the compressive strength to 42.4 ± 0.7 MPa compared with pure PCL (Fig. S2, Table S3) [3].

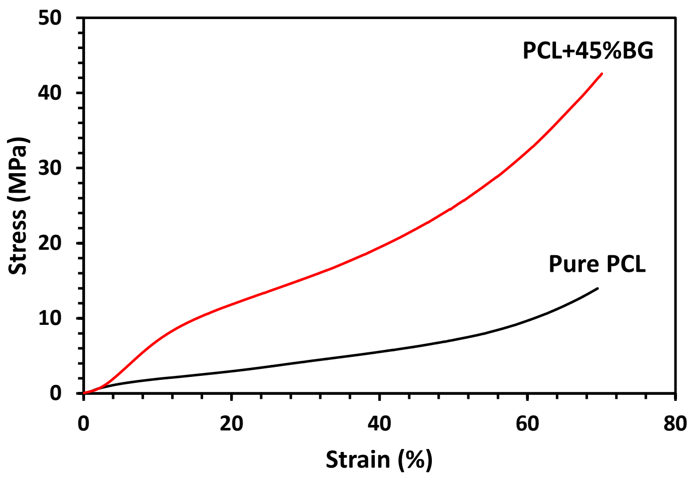


**Figure S2.** Compressive stress–strain curves of 3D-printed scaffolds composed of 45 wt.% 58S BG/PCL and pure PCL.

**Table S3.** Mechanical properties of the 45 wt.% 58S BG/PCL and pure PCL scaffolds.

| Scaffold composition | Compressive modulus (GPa) | Yield strength (MPa) | Compressive strength (MPa) |
|---|---|---|---|
| Pure PCL | 0.31 ± 0.03 | 3.6 ± 0.4 | 13.9 ± 0.4 |
| PCL+45%BG | 0.84 ± 0.04 | 9.1 ± 0.3 | 42.4 ± 0.7 |

The degradation profile of the 58S BG/PCL composite scaffold was evaluated through immersion in phosphate-buffered saline (PBS) at physiological temperature over 28 days. Mass measurements (Fig. S3a) and pH monitoring (Fig. S3b) were performed at predetermined intervals throughout the study period. The composite scaffold demonstrated minimal mass loss during the initial 5 days, followed by a progressive increase in degradation rate, culminating in approximately 18% weight reduction by day 28.

While pristine PCL scaffolds exhibited negligible degradation over the same duration, the incorporation of 58S BG nanoparticles significantly altered the degradation kinetics. The enhanced hydrophilicity imparted by the BG component facilitated fluid penetration and accelerated hydrolytic chain scission of the polymer matrix. Solution pH monitoring revealed an initial elevation from 7.4 to 7.9 within the first day, attributable to the release of calcium and silicate ions from the BG phase. Subsequently, the pH progressively decreased due to the accumulation of acidic degradation products from PCL hydrolysis, eventually stabilizing in the later stages of the experiment.

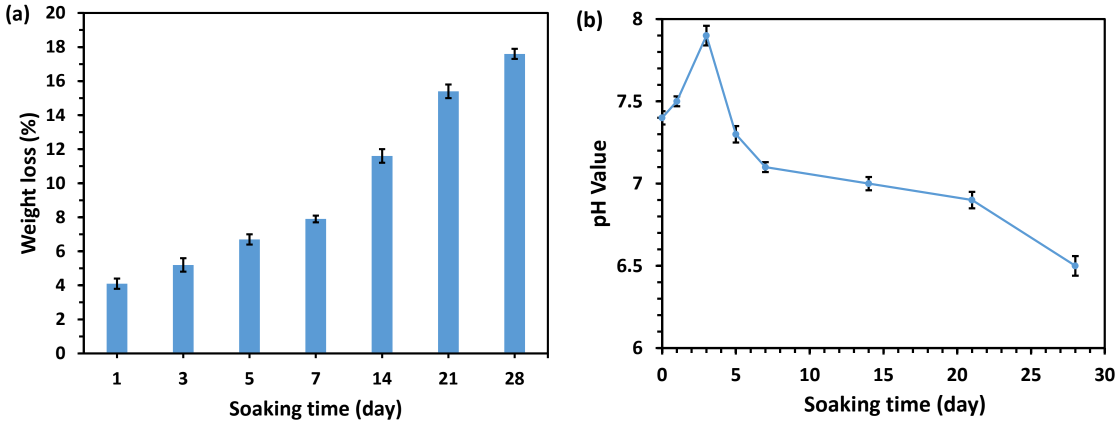


**Figure S3.** (a) Weight loss and (b) pH changes during immersion of 58S BG/ PCL scaffold samples in PBS at 37 °C for 28 days.

The bioactivity of the composite scaffold was evaluated by immersion in simulated body fluid (SBF) under physiological conditions for 28 days. Apatite formation was monitored using SEM, while solution pH and ionic concentrations were recorded throughout the incubation period. During the early days, a significant increase in pH was observed, attributed to the exchange of protons ($H_3O^+$) from the solution with $Ca^{2+}$ ions released from the bioactive glass (Fig. S4a). From day 3 to 28, the pH gradually decreased, reflecting the migration of $Ca^{2+}$ and $PO_4^{3-}$ ions toward the scaffold surface and the corresponding reduction of these ions in the solution. Inductively coupled plasma optical emission spectroscopy (ICP-OES) revealed a gradual depletion of calcium and phosphate ions from the SBF solution, indicating their consumption during apatite mineralization (Figs. S4b and S4c). In contrast, the silicon concentration in the solution continuously increased (Fig. S4d). Since SBF contains no silicon ions, the release of Si reflects the dissolution of the 58S BG component, serving as an indicator of scaffold biodegradability rather than contributing to apatite formation. Microstructural examination confirmed the formation of a continuous bone-like apatite layer on the scaffold surfaces after 14 days of immersion, with a notable increase in thickness observed by day 28 (Fig. S5). The mesoporous structure of the 58S BG component provided favorable nucleation sites for apatite crystallization, thereby accelerating the biomimetic mineralization process. X-ray diffraction (XRD) analysis (Fig. S6) showed increased crystallinity of the

deposited apatite over time, with characteristic hydroxyapatite peaks intensifying between 14 and 28 days.

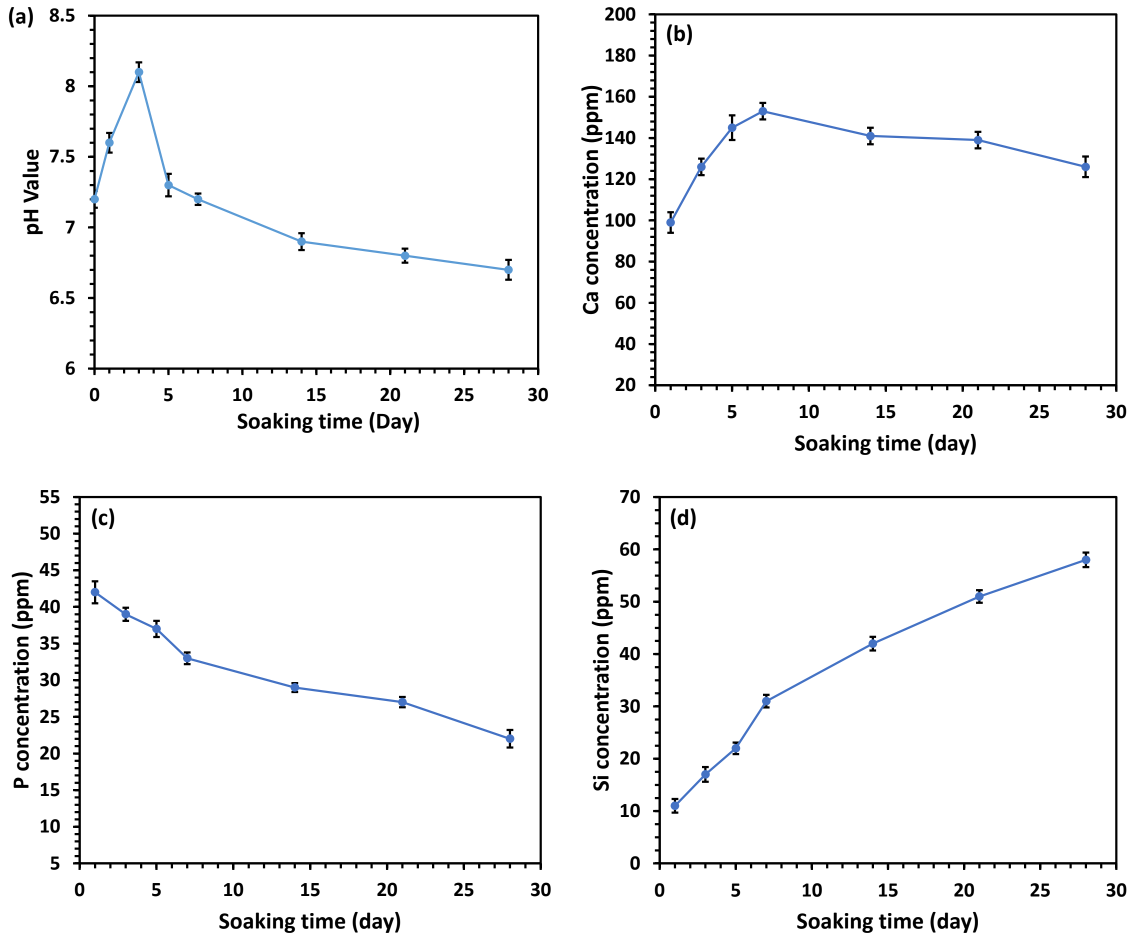


**Figure S4.** Changes in (a) pH and (b–d) concentrations of calcium, phosphate, and silicon ions in SBF during 28 days of immersion of 58S BG/PCL scaffold samples at 37 °C.

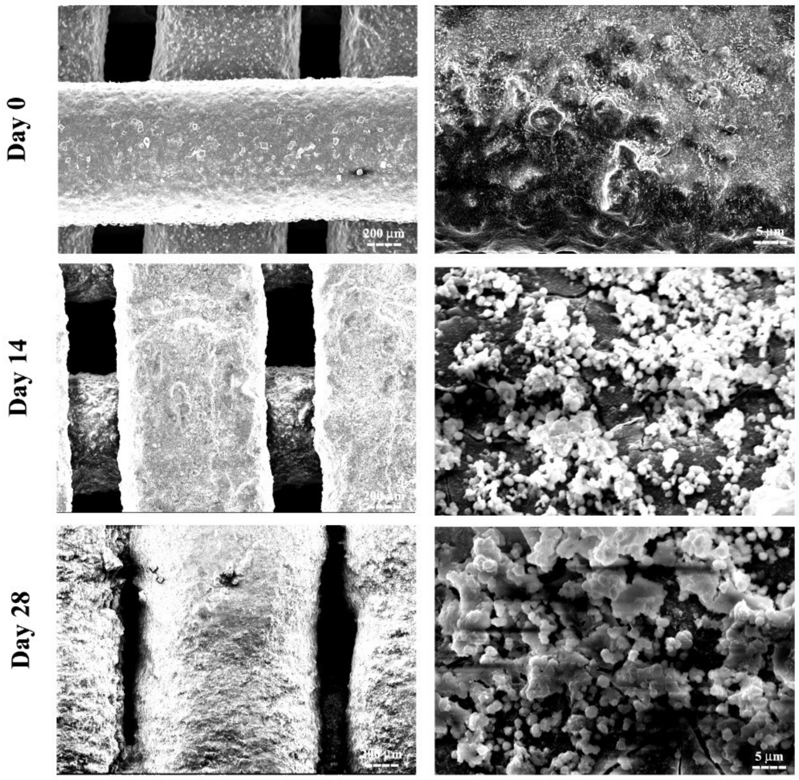


**Figure S5.** SEM micrographs of 58S BG/PCL scaffold surfaces after immersion in SBF for 0, 14, and 28 days at 37 °C.

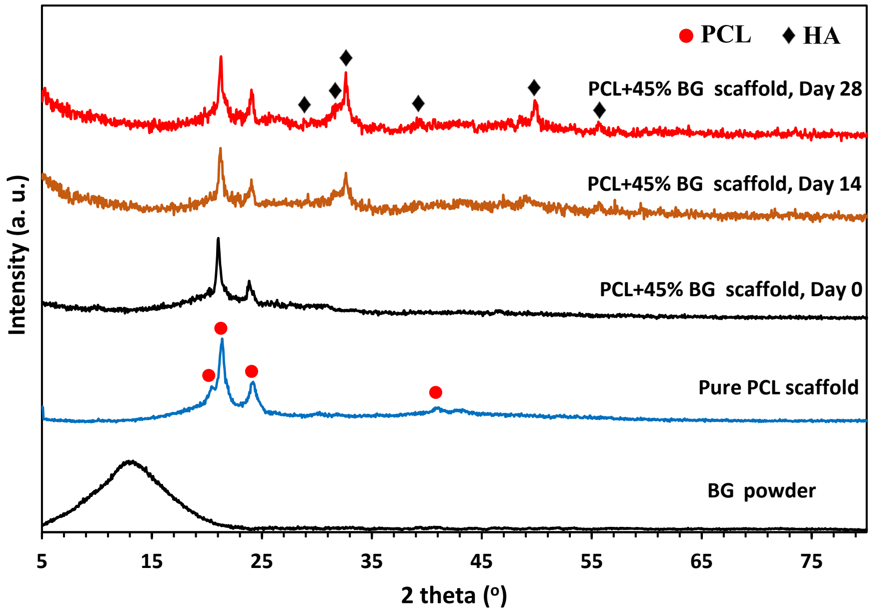


**Figure S6.** XRD patterns of 58S BG/PCL scaffold surfaces after immersion in SBF for 0, 14, and 28 days at 37 °C.

## 3. Synthesis and characterization of Maleimide-functionalized PCL

The alcohol functionalized furan-maleic anhydride adduct (FuMA) was synthesized according to previously reported work [4]. Maleimide-functionalized polycaprolactone (Mal-PCL) was synthesized through a two-step polymerization and deprotection strategy. Initially, FuMA was copolymerized with ε-caprolactone monomer using stannous octoate ($Sn(Oct)_2$) as a catalyst at 100°C under vacuum conditions. The resulting furan-protected polymer was subsequently purified through multiple precipitations in cold diethyl ether. The maleimide functionality was regenerated via thermal retro-Diels-Alder reaction by refluxing the protected polymer in toluene at 100°C for 6 hours. Following solvent removal and reprecipitation, the final Mal-PCL was obtained as a yellowish solid with an 86% yield. Structural confirmation was provided by $^1$H NMR spectroscopy (400 MHz, $CDCl_3$), revealing characteristic maleimide proton signals at 6.70 ppm alongside polycaprolactone backbone resonances: methylene protons adjacent to ester linkages at 4.05 ppm, α-methylene protons at 2.30 ppm, and backbone methylene protons between 1.64-1.37 ppm. Molecular weight determination via end-group analysis confirmed a number-average molecular weight ($M_n$) of 25,000 g/mol. This functionalized polymer provides reactive maleimide groups for subsequent covalent conjugation with thiol-containing biomolecules, including the Apt19s aptamer.

## 4. qRT-PCR Primer Information

Primers were designed using Primer-BLAST and synthesized commercially with HPLC purification. Primer specificity was verified by BLAST analysis. Melting curve analysis showed a single amplification peak for each primer pair, indicating specific amplification.

**Table S4.** Primer sequences and characteristics used for qRT-PCR analysis.

| Oligo Name Sequence 5' - 3' | Oligo ID | BC | Purif. | EC | MW (Da) | Tm (°C) | OD 260nm | nmol | Normalisation# µl required for 100µM solution |
|---|---|---|---|---|---|---|---|---|---|
| GAPDHF CTC TCT GCT CCT CCT GTT | 241029B016A04 16/25 | 18 | HPLC | 142,0 | 5,344 | 56 | 1,8 | 12,8 | 128 |
| GAPDHR ACG ACC AAA TCC GTT GAC | 241029B016B04 17/25 | 18 | HPLC | 175,0 | 5,453 | 54 | 2,2 | 12,8 | 128 |
| Runx2 F GCC TTC AAG GTG GTA GC | 241029B016C04 18/25 | 17 | HPLC | 161,0 | 5,226 | 55 | 1,9 | 11,6 | 116 |
| Runx2 R CGT TAC CCG CCA TGA CA | 241029B016D04 19/25 | 17 | HPLC | 158,0 | 5,115 | 55 | 3,0 | 18,7 | 187 |
| Ocn F AGG TAT CTG TGG GAG CT | 241029B016E04 20/25 | 17 | HPLC | 167,0 | 5,281 | 52 | 2,0 | 12,0 | 120 |
| Ocn R ATT GCT GCA CAC CTT C | 241029B016F04 21/25 | 16 | HPLC | 141,0 | 4,792 | 48 | 2,4 | 16,7 | 167 |
| Opn F GCC GAG GTG ATA GTC TGG TT | 241029B016G04 22/25 | 20 | HPLC | 194,0 | 6,204 | 60 | 2,2 | 11,4 | 114 |
| Opn R TGA GGT GAT GTC CTC GTC TG | 241029B016H04 23/25 | 20 | HPLC | 186,0 | 6,155 | 60 | 3,6 | 19,1 | 191 |
| ALP F GCA CCT GCC TTA CTA AC | 241029B016A05 24/25 | 17 | HPLC | 152,0 | 5,090 | 52 | 2,3 | 15,0 | 150 |
| ALP R AGA CAC CCA TCC CAT C | 241029B016B05 25/25 | 16 | HPLC | 150,0 | 4,755 | 51 | 2,2 | 14,7 | 147 |

**References**

[1] Bui, X.V. and Dang, T.H., 2019. Bioactive glass 58S prepared using an innovation sol-gel process. Processing and application of ceramics, 13(1), pp.98-103.
[2] Delpino, G.P., Borges, R., Zambanini, T., Joca, J.F.S., Gaubeur, I., de Souza, A.C.S. and Marchi, J., 2021. Sol-gel-derived 58S bioactive glass containing holmium aiming brachytherapy applications: a dissolution, bioactivity, and cytotoxicity study. Materials Science and Engineering: C, 119, p.111595.
[3] Soleymani, M., Moslemi, S., Dini, G., Ejeian, F. and Najafinezhad, A., 2026. ZIF-8 functionalized PCL/BG composite scaffolds with improved bioactivity and osteogenic differentiation. Scientific Reports (https://doi.org/10.1038/s41598-026-44943-1).
[4] Neubert, B.J. and Snider, B.B., 2003. Synthesis of (±)-phloeodictine A1. Organic Letters, 5(5), pp.765-768.
